# Photocatalytic Oxidation of Propylene on Pd-Loaded Anatase TiO_2_ Nanotubes Under Visible Light Irradiation

**DOI:** 10.1186/s11671-016-1486-6

**Published:** 2016-05-26

**Authors:** Chen Li, Lanlan Zong, Qiuye Li, Jiwei Zhang, Jianjun Yang, Zhensheng Jin

**Affiliations:** National & Local Joint Engineering Research Center for Applied Technology of Hybrid Nanomaterials, Henan University, Kaifeng, 475004 People’s Republic of China; Collaborative Innovation Center of Nano Functional Materials and Applications of Henan Province, Henan University, Kaifeng, 475004 People’s Republic of China

**Keywords:** Anatase TiO_2_ nanotubes, Visible light, Titanate nanotubes, Single-electron-trapped oxygen vacancies, Visible light photocatalysis

## Abstract

TiO_2_ nanotubes attract much attention because of their high photoelectron-chemical and photocatalytic efficiency. But their large band gap leads to a low absorption of the solar light and limits the practical application. How to obtain TiO_2_ nanotubes without any dopant and possessing visible light response is a big challenge nowadays. Orthorhombic titanic acid nanotubes (TAN) are a special precursor of TiO_2_, which possess large Brunauer-Emmett-Teller (BET) surface areas and strong ion exchange and adsorption capacity. TAN can transform to a novel TiO_2_ with a large amount of single-electron-trapped oxygen vacancies (SETOV) during calcination, while their nanotubular structure would be destroyed, and a BET surface area would decrease remarkably. And interestingly, SETOV can lead to a visible light response for this kind of TiO_2_. Herein, glucose was penetrated into TAN by the vacuum inhalation method, and TAN would dehydrate to anatase TiO_2_, and glucose would undergo thermolysis completely in the calcination process. As a result, the pure TiO_2_ nanotubes with visible light response and large BET surface areas were obtained. For further improving the photocatalytic activity, Pd nanoparticles were loaded as the foreign electron traps on TiO_2_ nanotubes and the photocatalytic oxidation efficiency of propylene was as high as 71 % under visible light irradiation, and the photostability of the catalyst kept over 90 % after 4 cyclic tests.

## Background

The last decades have witnessed the flying advance of our society; at the same time, many problems appear. One of the top problems is environmental concern. It has effects on ecological balance, human health, and sustainable development. Photocatalytic elimination of the organic pollutants is one of the efficient ways to alleviate the pollution of the environment and which has been widely studied in the past decades [1–5].

TiO_2_ has become the hottest photocatalyst since it was found by Fujishima in 1972 that TiO_2_ had the photoelectron-catalytic ability to make water splitting [6]. Since then, a TiO_2_ photocatalyst has attracted much attention and has been widely used in degradation of organic pollutants, CO_2_ reduction, and so on [7–12]. As is known to all, TiO_2_ can be excited by ultraviolet light, which leads to the low utilization of solar light. Besides, its photo-conversion efficiency is very low. To increase the light response region and enhance the efficiency, many approaches have been tried, such as modifying with metal or nonmetal ions [12–16], coupling with other narrow band gap semiconductors [17–19], or sensitizing by various dyes [20–23].

The titanate nanotube is a kind of TiO_2_-based material, which possesses a layered structure and a big Brunauer-Emmett-Teller (BET) surface area. The titanate nanotube has many advantages. Firstly, it has a tubular shape with a large surface area, which can provide more adsorption or active sites. Secondly, the layered structure and strong ion exchange ability is favorable for foreign atom doping or incorporating into the crystal lattice. Now, it has been widely used in many fields, such as photocatalysis and solar cells [24, 25]. Our group has systematically studied titanic acid nanotubes (TAN) in the previous work [26–29]. However, it is a pity that TAN itself does not possess photocatalytic activity, until it experiences a high temperature treatment or hydrothermal process, then it turns into a kind of novel TiO_2_. The novel TiO_2_ has many merits different from the normal TiO_2_. Single-electron-trapped oxygen vacancies (SETOV) appeared in the novel TiO_2_ when dehydration happens between the layers during the calcination process. SETOV would form a sub-band in the forbidden gap of TiO_2_, and as a result, this novel TiO_2_ could be excited by visible light [30–32]. However, it also has a natural defect, and the nanotubular structure of TAN will collapse during the dehydration process, resulting in a great decrease of the BET surface area. As well known, the BET surface area of a photocatalyst is very important for its photoactivity.

So how to take full advantage of these excellent merits and avoid the structure collapse of TAN are the key points. In our previous work, we use the “pillar-effect” method, through special measures to put small molecules into TAN, and then they will play a protective role in the tube when calcined. Previously, lanthanum nitrate was selected as a pillar to maintain the tubular morphology [33]. In the process of La(NO_3_)_3_ entering into the TAN, La^3+^ would exchange ions with the wall of the TAN and would incorporate into the crystal lattice of the TAN, so some La^3+^ ions cannot be removed completely. Although the high visible light-responded photocatalytic efficiency on the La-doped TiO_2_ nanotubes was achieved, the doped lanthanum would astrict its wide application as a pure anatase TiO_2_ precursor. Then we come up with small organic molecules as pillars, hoping to get pure anatase TiO_2_ nanotubes. Glucose was selected to substitute La(NO_3_)_3_ to prepare the anatase TiO_2_ nanotubes, and the ion exchange process between the incorporated solution and TAN was avoided. In the calcination process, glucose would be removed completely and the pure anatase TiO_2_ nanotubes can be obtained. Pd nanoparticles were further loaded as the electron traps on TiO_2_ nanotubes to improve the photocatalytic activity. The photo-oxidation removal efficiency of propylene was as high as 71 % under visible light irradiation. These pure anatase TiO_2_ nanotubes with a small diameter, large BET surface areas, and visible light response would have a large potential in the field of visible light photocatalysis and solar cell.

## Methods

### Preparation of the Pure Anatase TiO_2_ Nanotubes

**Scheme 1:**
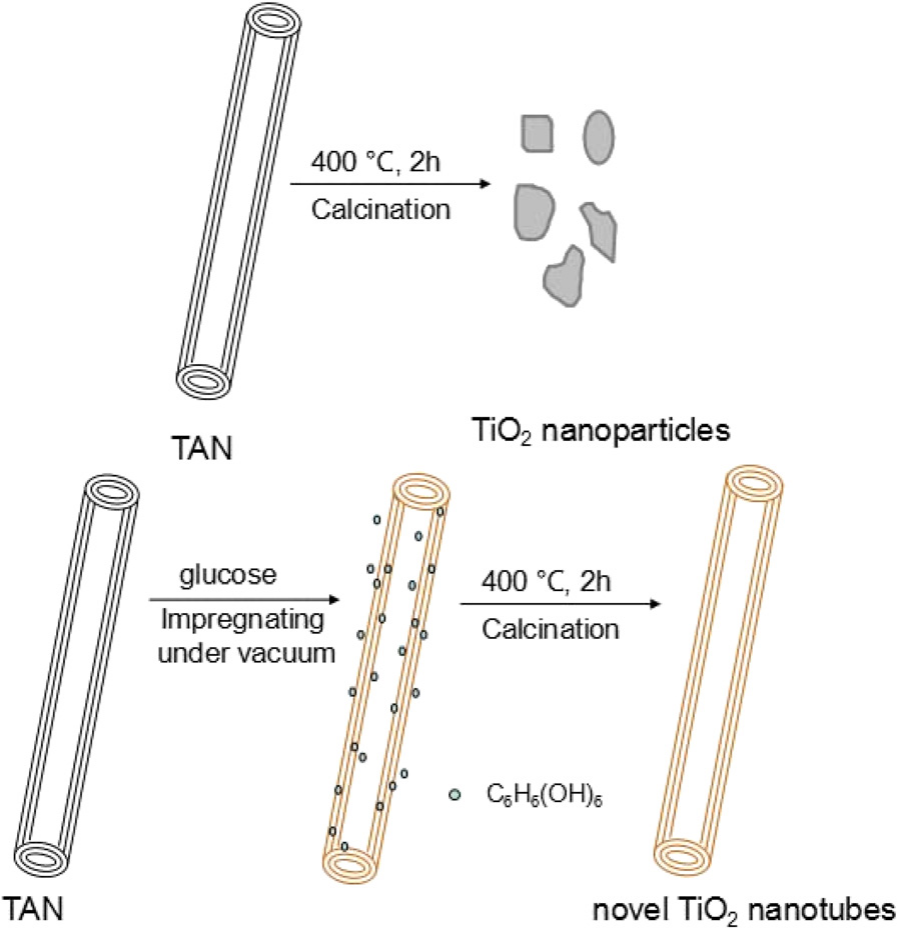
Formation diagrammatic sketch of TiO_2_ nanotubes

The details of the experiments were carried out in accordance with the following steps as simplified in Scheme 1. Firstly, TAN was prepared by the hydrothermal method reported in our previous work [26]. Then the TAN were soaked with ethanol for 24 h and followed by filtering and drying. After that, TAN was impregnated in 0.01 M glucose solution under vacuum to divert glucose into the nanotube, and then the residual organics of the nanotubes with purified water were cleaned out. At last, the product was calcined in air at 400 °C for 2 h, and as a result, the pure anatase TiO_2_ nanotube was obtained. For comparison, the TiO_2_ nanoparticles were obtained by calcining the TAN directly at 400 °C for 2 h. To improve the photocatalytic activity, 1 wt.% Pd nanoparticles were loaded as the electron traps on the surface of the TiO_2_ nanotubes, which was carried out by the photoreduction of 1 mmol/L PdCl_2_ in ethanol under UV light for 1 h.

### Evaluation of Photocatalytic Activity

The photocatalytic activity was evaluated by monitoring the oxidation of propylene under visible light irradiation. A 300-W Xe arc lamp with a 420-nm cutoff filter was used as the light source (*λ* ≥ 420 nm, *I* = 0.13 mW cm^−2^); meanwhile, a water cell was used to prevent the infrared light irradiation. Catalyst powder of 25 mg was spread on the surface of a roughened glass plate (10 cm^2^) located in a flat quartz tube reactor. Prior to light irradiation, the system was kept in dark for 2 h until reaching C_3_H_6_ adsorption-desorption equilibrium. The feed gas (flowing rate was kept at 150 mL h^−1^) was made up of pure C_3_H_6_ and Ar, which was stored in a high-pressure cylinder. The content of C_3_H_6_ was determined at a sensitivity of 1 ppmV by a chromatographic method on line analysis (Shimadzu GC-9A with a flame ionization detector, a GDX-502 column, and a reactor loaded with a Ni catalyst for the methanization of CO_2_); time interval for each analysis is 10 min. The degradation rate of C_3_H_6_ (*V*) = (*C*_0_ − *C*)/*C*_0_ × 100 %, in which *C*_0_ refers to the concentration of feed gas C_3_H_6_.

### Characterization of Samples

A transmission electron microscope (TEM), JEOL JEM-2010, with accelerating voltage of 200 kV, was applied to observe the morphology of the catalysts. The BET specific surface area of the samples was determined by N_2_ adsorption-desorption method with a Quadrasorb SI equipment (pretreatment 200 °C/6 h). X-ray diffraction (XRD) patterns were measured on a Philips X’Pert Pro X-ray diffractometer (Holland) (Cu *K*α radiation; 2*θ* range 15° ~ 85°; step size 0.08°; time per step 1.0 s; accelerating voltage 40 kV; applied current 40 mA). Raman shift was recorded with a Raman spectroscopy (RM-1000 Renishaw), with a wavelength range of 100–1750 cm^−1^. UV-vis diffuse reflectance spectroscopy (DRS) was carried out in diffuse reflection pattern with a spectrophotometer (Lambda 950, Perkin Elmer) equipped with a wavelength range of 200–800 nm (BaSO_4_ as a reference). Electron spin resonance (ESR) spectra were measured on a Bruker E500 spectrometer at room temperature in ambient air (without supplied vacuum). The catalysts of X-ray photoelectron spectroscopy (XPS) data were obtained through a Kratos AXIS Ultra spectrometer (excitation source: monochromatized Al *K*α (hν = 1486.6 eV); current 10 mA; voltage 15 kV). The binding energies were normalized to the signal for adventitious hydrocarbon at 284.8 eV.

## Result and Discussion

### Formation Mechanism and Structure of TiO_2_ Nanotubes

**Fig. 1:**
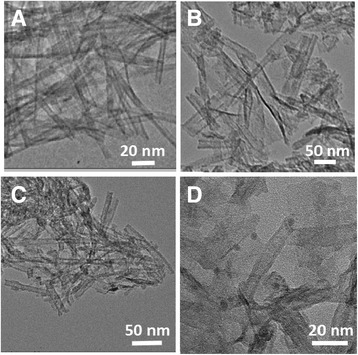
TEM images of the photocatalysts obtained. **a** TAN. **b** TiO_2_ nanotubes. **c** TiO_2_ nanoparticles. **d** Pd-loaded TiO_2_ nanotubes

The TAN can dehydrate to form anatase TiO_2_ under calcination. But the one-dimensional nanotubular structure would collapse, and the BET surface areas will decrease remarkably, as shown in Scheme 1. From our previous work, we knew that this kind of TiO_2_ possessed a large amount of SETOV that led to a visible light response. How do the nanotubular structure, large BET surface area, and the visible light absorption of this kind of TiO_2_ remain? Herein, glucose was selected as the pillar of the nanotube structure, which was soaked into TAN under high vacuum. When calcining a TAN/glucose composite, TAN would dehydrate to anatase TiO_2_, and glucose would decompose completely, and as a result, a pure TiO_2_ nanotube without any dopant was obtained. The TEM images in Fig. 1 verified this idea of preparation method successfully. Figure 1a shows that the TAN showed a very uniform nanotube morphology and their diameters were ca. 8–10 nm. Figure 1b illustrates that the sample uses glucose as the pillar support and the integrity of the nanotubes implied that glucose was indeed playing the important role of protecting the nanotubular structure. At the same calcination conditions, the tubular shape has partially collapsed and a little part of the short tubes and most nanoparticles left without glucose as the support (as shown in Fig. 1c). Figure 1d illustrates that the layered nanotubular morphology was kept very well after loading the Pd nanoparticles. Palladium existed not only on the surface of the TiO_2_ nanotubes but also in the inner of the nanotubes. The average particle sizes of the Pd nanoparticles were ca. 3 nm.

**Fig. 2:**
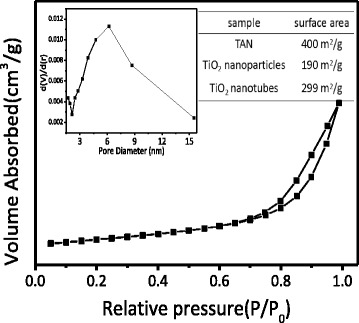
Nitrogen adsorption-desorption isotherms and pore size distribution curves (*inset*) of as-prepared TiO_2_ nanotubes obtained

Figure 2 shows a typical example of the nitrogen adsorption-desorption isotherms of TiO_2_ nanotubes. The sharp decline in the desorption curve and the hysteresis loop at high relative pressure are indicative of mesoporosity. As seen from the inset of Fig. 2, the pore size of TiO_2_ was in the range of 4–8 nm. The BET specific surface area of TiO_2_ nanotubes was 299 m^2^g^−1^, which increased apparently compared to that of the TiO_2_ nanoparticles (*S*_BET_ = 190 m^2^g^−1^) obtained by calcination of TAN directly in the air at 400 °C. The increased BET surface area should be due to the good nanotubular morphology of TiO_2_. The adsorption-desorption isotherm contained an obvious H_2_-type hysteresis loop with a highly delayed desorption branch resulted from the hollow structure of the nanotubes, which were in accordance with the HRTEM images of Fig. 1. Furthermore, the BET surface area of the pure TiO_2_ nanotubes decreased a little compared with the as-prepared TAN, which should be due to the broken part of the nanotubes.

**Fig. 3:**
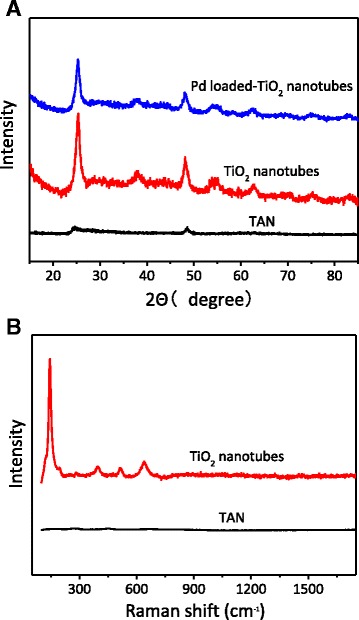
XRD patterns (**a**) and Raman spectra (**b**) of TAN and Pd-loaded TiO_2_ nanotubes obtained

The phase structure of the photocatalysts was measured by XRD and Raman techniques. The XRD pattern showed that TAN belong to the orthorhombic structure, consistent with the layered titanate (JCPDS No. 47-0124) in the previous reports [29–32]. When calcined at 400 °C, the TAN transformed to anatase TiO_2_ completely and the diffraction peaks at about 2*θ* = 25.37°, 37.88°, 48.12°, 53.79°, 55.10°, 62.74°, and 68.79° were assigned to the (101), (004), (200), (105), (211), (204), and (116) crystal faces of anatase, respectively [33]. There is no apparent peak detected belonging to palladium, probably because it had a uniform dispersion and the loading amount was rather low. The Raman spectra shown in Fig. 3b also confirmed the results of XRD, and the TAN has transformed to the pure anatase phase completely. And there is no organic residue on the surface, indicating that glucose had decomposed completely [34].

**Fig. 4:**
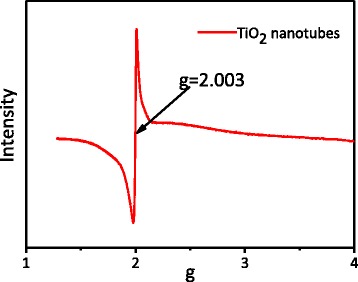
ESR spectra of TiO_2_ nanotubes obtained in ambient air

In our previous researches [28, 29], we found that when TAN were dehydrated at 400 °C or above, a novel anatase TiO_2_ containing a large amount of SETOV was obtained. Such a novel TiO_2_ contains a high concentration of intrinsic defects in the bulk, but its surface still retains stoichiometric structure. In addition, a high concentration of SETOV is favorable for the formation of a sub-band within the forbidden band of TiO_2_. As a result, such a novel TiO_2_ can be excited by visible light. In this work, whether the TiO_2_ nanotubes prepared from TAN through the pillar effect still possess this kind of SETOV is a question. As can be seen from the ESR spectra in Fig. 4, the present signal at 2.003 is of character peak of SETOV, indicating that the glucose-supported sample contains a certain amount of SETOV, which would help increase the absorption of the visible light region and further be favored to enhance the photocatalytic efficiency.

**Fig. 5:**
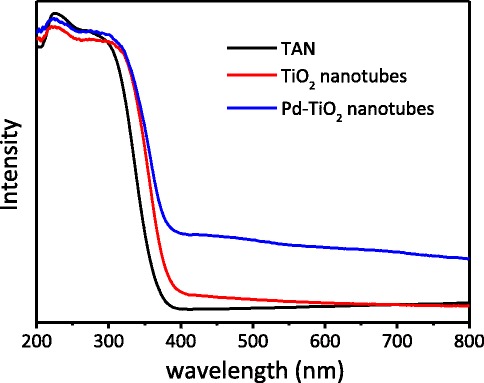
UV-vis diffuse reflectance spectra of as-prepared TAN, TiO_2_ nanotubes, and Pd-loaded TiO_2_ nanotubes obtained

UV-vis diffuse reflection spectra of the samples were illustrated in Fig. 5. The absorption onset wavelength of TAN is ca. 370 nm, and the energy band gap was calculated to be 3.35 eV. When TAN transformed to TiO_2_ nanotubes, the absorption redshifted apparently, which was in accordance with TEM and XRD results. The broad shoulder peak at ca. 400–450 nm in the visible light region should be due to the sub-band formed by SETOV in the band gap of the TiO_2_ nanotubes [28]. After loading the Pd nanoparticles on the TiO_2_ nanotubes, the visible light absorption enhanced remarkably; this phenomenon should be due to the plasma resonance absorption of the noble metal palladium [35]. This high visible light absorption should be helpful to enhance photocatalytic activity.

**Fig. 6:**
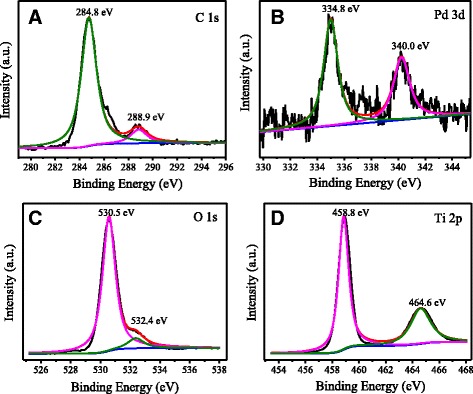
XPS spectra of Pd-loaded TiO_2_ nanotubes obtained: **a** C1s, **b** Pd 3d, **c** O1s, and **d** Ti 2p

The chemical composition and valence state of the surface elements of the Pd-loaded-TiO_2_ nanotubes were analyzed by XPS measurement. The chemical states of C1s, Pd 3d, O1s, and Ti 2p species were obtained by analyzing the XPS core levels shown in Fig. 6. The C1s peaks at 284.8 and 288.9 e V were assigned to the contaminative hydrocarbon adsorbed on the sample surface. These results implied that there are no other organic residues remaining on the surface of the catalyst. The binding energies for Pd 3d_5/2_ and Pd 3d_3/2_ that appeared at 334.8 and 340.0 eV, respectively, explained that the valence state of palladium was Pd^0^ [35–38], indicating that the Pd nanoparticles were loaded on the TiO_2_ nanotubes successfully. The chemical state of oxygen and titanium are the same with that of the TiO_2_ nanoparticles. The peaks at 532.4 and 530.5 eV were corresponding to the crystal lattice oxygen and adsorbed oxygen. Two characteristic peaks of Ti2p that appeared at 458.8 and 464.6 eV in Fig. 6d were indexed to Ti 2p_1/2_ and Ti 2p_3/2_, respectively, indicating that titanium is entirely at presence of Ti^4+^.

**Fig. 7:**
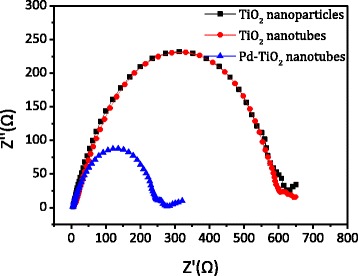
Electrochemical impedance spectroscopy of TiO_2_ nanotubes, TiO_2_ nanoparticles, and Pd-loaded TiO_2_ nanotubes

Electrochemical impedance spectroscopy is helpful to probe the features of surface-modified electrodes. And an EIS spectrum often displays the conductivity of an electrode, and a larger arc radius usually shows a higher charge transfer resistance. So the electron transport properties of the TiO_2_ nanoparticles, TiO_2_ nanotubes, and 1 % Pd-TiO_2_ nanotubes were characterized by EIS and shown in Fig. 7. And the Nyquist plots of the EIS spectra were measured in 0.1 M KOH aqueous solution under visible light irradiation (*λ* ≥ 420 nm, *I* = 0.13 mW cm^−2^). The arc radii of the TiO_2_ nanoparticles and TiO_2_ nanotubes were similar, but they are much larger than that of the 1 % Pd-TiO_2_ nanotube electrode. That indicated that the 1 % Pd-TiO_2_ nanotubes displayed a much higher separation efficiency of photo-generated electron and hole pairs, which would be beneficial to the improvement of its photocatalytic activity. Therefore, doping the Pd nanoparticles in the TiO_2_ nanotubes has a significant impact on the photoelectron conversion efficiency.

### Photo-oxidation Removal of Propylene on Pd-Loaded TiO_2_ Nanotubes Under Visible Light Irradiation

**Fig. 8:**
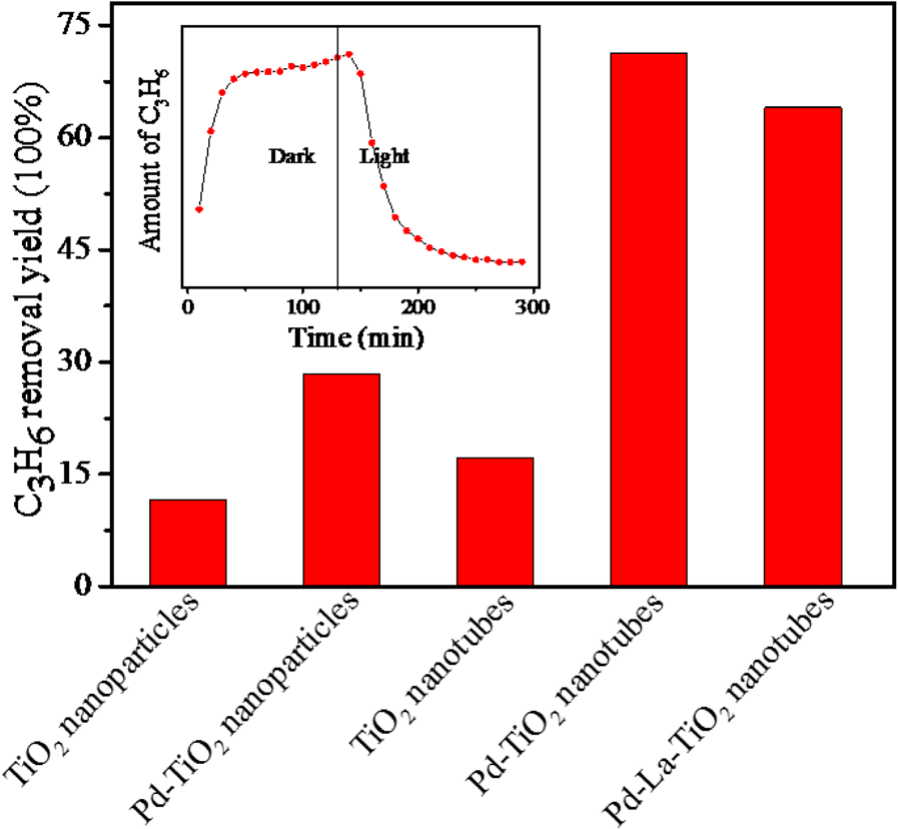
The yield of photo-oxidation removal of propylene on all samples under visible light

The photocatalytic activities of the catalysts were evaluated by monitoring oxidation of propylene under visible light irradiation. As can be seen in Fig. 8, the degradation yield of C_3_H_6_ on the TiO_2_ nanotubes was 17 %, which was much higher than 11 % of the TiO_2_ nanoparticles. The reason should be due to the larger BET surface area and one-dimensional nanotubular morphology of TiO_2_ nanotubes. After loading the Pd nanoparticles, the photocatalytic activity increased largely and removal yield of C_3_H_6_ on the Pd-loaded TiO_2_ nanotubes reached 71 %. After loading the Pd nanoparticles, the visible light absorption increased apparently than the bare one, indicating that the utilization efficiency of the incident light increased accordingly. In addition, as a good foreign electron trap, Pd can transfer the photo-generated electrons of the TiO_2_ nanotubes, thereby improving the separation efficiency of the charge carriers, so a higher photo-oxidation efficiency of C_3_H_6_ was obtained. Moreover, comparing with the Pd-loaded La-doped TiO_2_ nanotubes in our former work, we found that the photoactivity of the Pd-loaded TiO_2_ nanotubes was better than that of the La-doped samples. The reason can be explained as that the La dopant may destroy some lattice of TiO_2_ and form a lattice defect, which would become the recombination of the photocharge carriers. From the above analysis, we can conclude that the pure TiO_2_ nanotubes with large BET surface areas, visible light absorption, and visible light-responded photocatalytic activity have been fabricated from NTA assisted with the pillar effect of glucose.

**Fig. 9:**
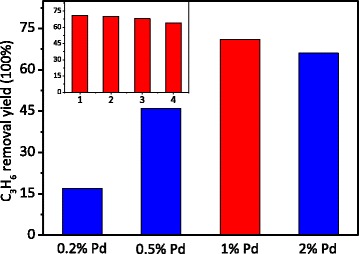
The yield of photo-oxidation removal of C_3_H_6_ on different amounts of Pd-loaded TiO_2_ nanotubes; photostability test of 1 % Pd-TiO_2_ nanotubes displayed in the *inset*

The effect of loading amount of the Pd nanoparticles on photo-removal efficiency of propylene was investigated in Fig. 9. As can be seen, as the loading amount of Pd increases from 0.2 to 2 %, the removal efficiency of propylene first increased and then decreased. Too little amount of Pd could not trap enough photo-generated electrons to realize the best separation of the charge carriers, while too much amount of Pd would agglomerate and become the recombination centers of the electrons and holes. And as a result, the best photoactivity was obtained as high as 71 % on the sample of the 1 % Pd-loaded TiO_2_ nanotubes. Moreover, the photostability of the optimum photocatalysts for propylene removal was evaluated. As shown in the inset of Fig. 9, after four times of circulation experiment, the C_3_H_6_ removal yield on the 1 % Pd-TiO_2_ nanotubes was also kept 64 %, indicating that the photocatalytic efficiency remained over 90 % after a long-term reaction, which testified that the stability of the photocatalyst was excellent.

## Conclusions

The pure TiO_2_ nanotubes with excellent visible light photoactivity were prepared using TAN as the precursor and glucose as a pillar support. During calcination, TAN would dehydrate into the anatase TiO_2_, and glucose went through thermolysis completely. The BET surface area was as large as 299 m^2^ g^−1^, which was much larger than that of the TiO_2_ nanoparticles obtained by direct calcination of TAN. The Raman and XPS results proved that there were no organic residues left on the surface or in the phase of the TiO_2_ nanotubes. The ESR spectra implied the existence of a large amount of SETOV. When the Pd nanoparticles were loaded on the TiO_2_ nanotubes, the photo-oxidation removal efficiency of propylene was as high as 71 % under visible light irradiation, which should be attributed to the higher separation efficiency of the photo-generated electrons and holes, the large BET surface area, and strong visible light absorption of the materials. These pure anatase TiO_2_ nanotubes with a small diameter, large BET surface areas, and visible light response would have a large potential in the field of visible light photocatalysis and solar cells.
